# Economic evaluation of Cytosponge®-trefoil factor 3 for Barrett esophagus: A cost-utility analysis of randomised controlled trial data

**DOI:** 10.1016/j.eclinm.2021.100969

**Published:** 2021-06-18

**Authors:** Nicholas Swart, Roberta Maroni, Beth Muldrew, Peter Sasieni, Rebecca C. Fitzgerald, Stephen Morris, BEST3 Consortium

**Affiliations:** a Department of Applied Health Research, University College London, UK; b School of Cancer and Pharmaceutical Sciences, Cancer Research UK & King's College London Cancer Prevention Trials Unit, Cancer Prevention Group, King's College London, UK; c BEST3 Trial team NIHR, Clinical Research Networks, UK. Full list of members given in Appendix, UK; d Cambridge University Hospitals NHS Foundation Trust, UK; e MRC Cancer Unit, Hutchison-MRC Research Centre, University of Cambridge, UK; f Primary Care Unit, Department of Public Health and Primary Care, University of Cambridge, UK

**Keywords:** Screening, Cancer prevention, Early detection, Esophagus, Neoplasia

## Abstract

**Background:**

Esophageal adenocarcinoma has a very poor prognosis unless detected early. The Cytosponge-trefoil factor 3 (TFF3) is a non-endoscopic test for Barrett esophagus, a precursor of esophageal adenocarcinoma. Randomised controlled trial data from the BEST3 trial has shown that an offer of Cytosponge-TFF3 in the primary care setting in England to individuals on medication for acid reflux increases detection of Barrett esophagus 10-fold over a year compared with standard care. This is an economic evaluation of Cytosponge-TFF3 screening versus usual care using data from the BEST3 trial which took place between 20th March 2017 and 21st March 2019.

**Methods:**

A Markov model with a one-year cycle-length and a lifetime time horizon was created, adapting previous modeling work on Cytosponge screening. The impact of one round of Cytosponge screening was modelled in patients with a median age of 69 years (based on BEST3 trial population). Cost-effectiveness was expressed as an incremental cost-effectiveness ratio (ICER). Deterministic and probabilistic sensitivity analyses were conducted on model parameters.

**Findings:**

Per person, one round of Cytosponge-TFF3 screening, including confirmatory endoscopy and treatment, in the intervention arm costed £82 more than usual care and generated an additional 0.015 quality-adjusted life-years (QALYs) at an ICER of £5,500 per QALY gained. Probabilistic sensitivity analysis gave an ICER of £5,405 (95% CI -£6,791 to £17,600). The average QALY gain per person is small because the majority of patients in the model will not develop BE and therefore will have no resulting change in their utility, however the small proportion of patients who are identified with BE dysplasia or cancer derive large benefit. At a willingness-to-pay threshold of £20,000 per QALY, the probability that Cytosponge-TFF3 was cost-effective was over 90%.

**Interpretation:**

Using data from a pragmatic randomised trial, one-off Cytosponge-TFF3 screen is cost-effective relative to usual care for patients with gastro-esophageal reflux disease, despite relatively low uptake and an older population in this trial setting than previously modelled. Improving Cytosponge-TFF3 uptake and targeting younger patients is likely to further improve cost-effectiveness.

**Research in context**

**Evidence before this study**

Barrett esophagus is a pre-cancerous condition which, if diagnosed, can permit early detection and curative treatment of dysplasia and esophageal adenocarcinoma. Randomised controlled trial data from the BEST3 study has shown that offering a novel non-endoscopic test, the Cytosponge-trefoil factor 3 (TFF3), in the primary care setting in England can diagnose ten times more Barrett esophagus than usual care over a year. The cost-effectiveness of this trial is not known.

**Added value of this study**

This economic model builds upon modeling used by Benaglia et al. 2013 that used a hypothetical cohort. Our analysis amended and adapted that model and applied it to clinical trial data collected from the BEST3 trial. This analysis suggests that the Cytosponge-TFF3 test is cost-effective in a real world setting and could be adopted at a lower threshold for willingness-to pay per QALY gained than previously estimated. In the trial setting patients were older with a lower uptake rate with consequences for cost-effectiveness estimation, improving uptake and targeting younger patients would add further benefit.

**Implications of all the available evidence**

The published evidence suggests that the Cytosponge-TFF3 procedure is cost-effective and affordable if provided as a triage test for people with gastro-esophageal reflux disease to increase detection of Barrett esophagus. Although systematic Cytosponge-TFF3 testing for individuals on medication for reflux incurs higher costs per person than usual care, and involves additional diagnostic endoscopy in a minority, Cytosponge-TFF3 also generates additional quality-adjusted life-years due to earlier cancer diagnosis and curative treatment.

Alt-text: Unlabelled box

## 1 Introduction

The incidence of esophageal adenocarcinoma (EAC) has increased six-fold in northwest Europe, North America, Australia, and New Zealand since the 1990s [1,2], making esophageal cancer a source of significant public health concern. The overall 5-year survival is less than 20% across multiple high income countries worldwide [3]. One of the key factors leading to poor outcomes is the late stage at presentation [4]. Reflux symptoms such as recurrent and severe heartburn are a major risk factor which increases the risk of developing EAC, and since reflux is highly prevalent (estimates range between 12% to 40% of adults) [5], it is challenging to devise a feasible large-scale diagnostic and prevention strategy. However, the presence of a pre-malignant precursor lesion to EAC, called Barrett esophagus (BE), provides an opportunity to identify a high-risk population so that intervention can be more targeted. There have been significant advances in cost-effective, outpatient-based endoscopic therapies which are now recommended for low- and high-grade dysplasia as well as intramucosal stage-1 cancer in BE with very low rates of recurrence [6], [7], [8]. These treatment advances substantially mitigate the risks and side effects from systemic therapy and esophagectomy required for more advanced disease [9,10].

The major challenge remains identifying individuals with BE, since using current clinical guidelines, it is estimated that only 20% of BE is diagnosed and hence the majority of EAC cases are diagnosed *de novo* without the opportunity to prevent progression [11,12]. Endoscopy for all individuals with reflux symptoms would be costly and pose a logistical challenge for the health system. To overcome this problem and enable diagnostic triage in primary care, there is considerable interest in developing non-endoscopic approaches [13]. Cytosponge-TFF3 is a non-endoscopic cell collection device coupled with a laboratory test for the specific biomarker Trefoil Factor 3 (TFF3), which identifies intestinal metaplasia, i.e. the histopathological hallmark of pre-malignant BE. Two clinical studies have previously been carried out, which have demonstrated the safety, acceptability and performance characteristics of this test [14]. Recently, a large pragmatic, randomised, controlled trial (BEST3), involving 13,657 patients with recurrent reflux symptoms who were on acid-suppressant medication prescribed by their General Practitioner in England has been reported [15]. This trial showed that the Cytosponge-TFF3 test administered in the primary care setting, followed by a confirmatory endoscopy if the Cytosponge-TFF3 result was positive (13%), leads to a substantial increase in BE cases identified. Attendance rates for research interventions are commonly challenging. However, despite only 24% (1654 out of 6834) of patients in the intervention arm attending to receive this test in this research setting, ten times more patients were diagnosed with BE in the intervention than in the usual care arm over 12 months follow-up (in intention-to-treat analysis and rate ratio after adjustment for cluster randomization 10.6; 95% CI 6.0–18.8; *p* < 0.001). In those who underwent the Cytosponge-TFF3 procedure, 131 participants (8% of the 1654 patients who swallowed a Cytosponge-TFF3 and 59% of the 221 patients receiving a confirmatory endoscopy following a positive Cytosponge-TFF3 results) had BE or cancer diagnosed. Esophago-gastric neoplasia diagnoses were a secondary endpoint of the trial. Although the numbers were small, the offer of Cytosponge-TFF3 led to an increased detection of early neoplasia (dysplastic Barrett esophagus or stage I cancer) compared with the control arm (9 vs. 0).

**Fig. 1:**
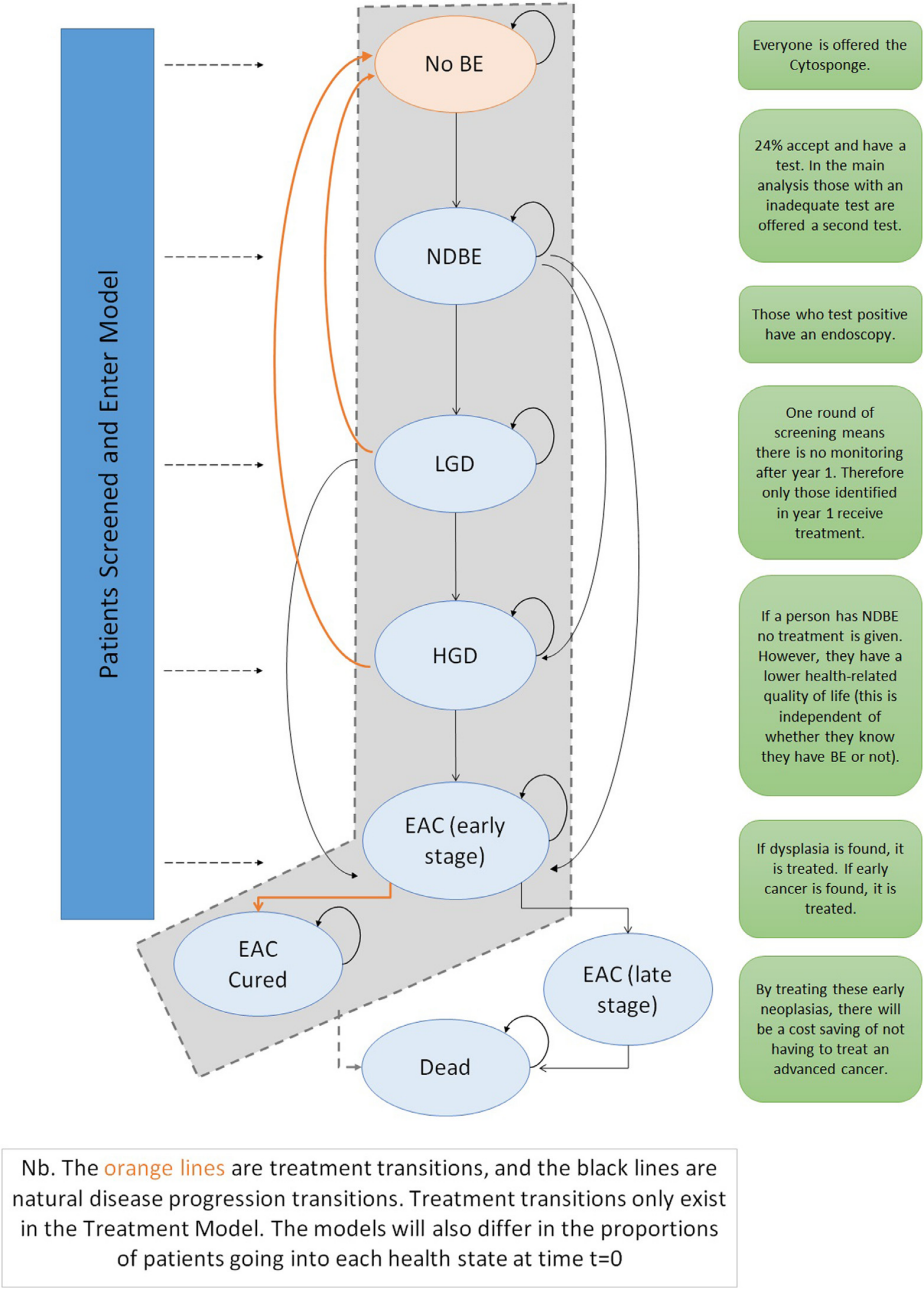
Markov model with transitions for treatment and for natural history (no treatment) patients moving between disease states. BE = Barrett esophagus; NDBE = non-dysplastic Barrett esophagus; LGD = low-grade dysplasia; HGD = high-grade dysplasia; EAC = esophageal adenocarcinoma.

Health economic evaluation is an essential part of implementation of new diagnostic tests, by modeling their impact compared with standard care (see Fig. 1). Previous economic evaluations of Cytosponge screening have been favourable but have relied on estimates from previous cohort studies without the availability of randomised trial evidence to populate the model [16,17]. Using the results of the BEST3 trial, we conducted a cost-utility analysis of offering Cytosponge-TFF3 testing for patients on long-term treatment with acid suppressants for gastro-esophageal reflux disease (GERD) compared with the current standard of care.

## 2 Methods

### 2.1 Design and overview

We undertook a detailed analysis of the costs and cost-utility of the BEST3 trial comparing Cytosponge-TFF3 screening (with a confirmatory endoscopy for positive TFF3 patients) with the current standard of care, which entails treatment of heartburn-predominant symptoms and referral for endoscopy as deemed necessary by the primary care physician. Consistent with the trial design this considered one round of Cytosponge-TFF3 screening in a pre-defined cohort, followed up for one year. We used Markov chains to model disease state progression for BE through to late-stage EAC, which were adapted from a previously developed decision analytic model [16] concerning Cytosponge-TFF3 screening. We did not consider multiple rounds of screening because the incidence of BE in a cohort that has already been screened by Cytosponge-TFF3 is not yet known. The cost of screening was calculated based on the starting proportion of patients and the uptake rate of Cytosponge-TFF3 in the cohort. We used a lifetime time horizon from the perspective of the UK National Health Service (NHS), and a discount rate of 3.5% as per the National Institute for Health and Care Excellence (NICE) guidelines [18]. Cycle length in the model was one year. Cost-effectiveness was measured in terms of an incremental cost-effectiveness ratio (ICER)-the incremental cost per quality-adjusted life-year (QALY) gained.

### 2.2 Study population values used in the economic analysis

In the BEST3 trial, Cytosponge was offered to individuals aged 50 years and over. However, there was a wide range of participation across all age groups with a median age of 69 years. Therefore, we used 69 years as the starting age of the cohort modelled in this economic evaluation. 6834 patients were enrolled in the intervention arm (in the intention-to-treat analysis) and offered the Cytosponge-TFF3 screening by a written invitation; 2679 (39%) expressed interest in taking part. Of these, 1750 were eligible, consented and attended for Cytosponge-TFF3, following which 1654 (95% of those who attended and 24% of all those in the intervention arm) successfully swallowed the device, comprising 796 men (48%) and 858 (52%) women. 311 (19%) of the 1654 participants were offered a repeat Cytosponge-TFF3 test due to an equivocal or low-confidence result (meaning gastric columnar cells were not present so could not guarantee that the distal esophagus had been sampled). Depending on local capacity and patient preference, 202 participants attended a second appointment, of whom 190 (94%) successfully swallowed the Cytosponge-TFF3. There was one serious adverse event associated with the Cytosponge-TFF3 test (detachment of the sponge from the thread requiring endoscopy to retrieve it) and sore throat was the commonest side-effect (4%). The base-case analysis compared the cost-effectiveness of Cytosponge-TFF3 in a cohort of 6834 patients taking these factors into account.

### 2.3 Model structure and disease prevalence

The structure of the Markov model used is shown in Fig. 1. At time *t* = 0, patients enter either the Treatment or Natural history model and the costs of screening are applied. The number of patients starting in each state is given in Table 1. All patients in the Treatment model receive treatment for BE, and successful patients transition to the “No BE” (No Barrett esophagus) state. Patients identified as true positives enter the Treatment model, all other patients including any false negatives from Cytosponge enter the Natural history model. False positive patients incur the cost of screening but no BE treatment costs. The prevalence of BE in this cohort was estimated at 9%.

**Table 1:** Starting numbers of patients entering model at different stages of disease identified by the BEST3 trial (under the assumption that *n* = 6834 for both the intervention and the usual care arms).

State	Intervention arm		Usual care arm	
	Treatment model	Natural history model	Treatment model	Natural history model
No BE	0	6230.6	0	6230.6
NDBE	123	443.6	11.6	555
LGD	1	3.6	0	4.6
HGD	3	10.8	0	13.8
Early EAC	4	14.4	4	14.4
Late EAC	0	0	0	0

BE = Barrett esophagus; NDBE = non-dysplastic Barrett esophagus; LGD = low-grade dysplasia; HGD = high-grade dysplasia; EAC = esophageal adenocarcinoma.

The sensitivity and specificity of Cytosponge was taken from the BEST2 trial of 79.9% and 92.4% respectively (not shown) [19]. This trial was designed to derive accuracy data and the sensitivity used is from a per protocol analysis that includes inadequate samples without a repeat examination to provide a conservative, base case. We assumed that the confirmatory endoscopy with biopsy that follows a positive TFF3 test result had an effective sensitivity and specificity of 1 (gold-standard) for the purposes of the model, in line with assumptions taken by previous economic models [16,17,20]. This approach takes into account the face validity of a negative endoscopy test in that clinicians typically do not re-order endoscopy following negative findings, even though the sensitivity of endoscopy is less than 100%. Therefore, we are in effect modeling “endoscopy detectable BE”. Half-cycle correction was applied. See Appendix for further details on methods.

Treatment data for PPI drugs and patients with EAC was taken from trial data. Late-stage cancer was treated with palliative care. Utilities were assigned regardless of whether any disease had been identified. Key model outputs are given in Table 2.

**Table 2:** Key model outputs, showing number of patients in the model who received screening, developed Barrett esophagus, and who developed and died from esophageal adenocarcinoma Nb.

Model outputs	Intervention arm (base case analysis)	Intervention arm (alternative scenario)	Usual care arm
	Cytosponge uptake at 24%	Cytosponge Uptake at 50%	
Number invited for Cytosponge-TFF3 screening	6834	6834	0
Number who had Cytosponge-TFF3 test	1654	3417	0
Number who had endoscopy	198	457	16
Number who start with or develop LGD*	344	321	343
Number who start with or develop HGD	143	123	151
Number who start with or develop early EAC	162	131	177
Number who die of EAC	153	112	173

⁎ The intervention arm had a slightly higher number of LGD cases because a) more patients were treated for LGD in the intervention arm; and b) patients who were treated for LGD returned to ‘No BE’, and therefore had a chance of getting worse again and progressing to NDBE and then LGD (and so on).

### 2.4 Costs and utilities

The costs of testing using the Cytosponge-TFF3 are high estimates based on introducing Cytosponge on a limited adoption basis since there is currently no National Schedule for this test. These include the device and centralised laboratory processing, the TFF3 antibody, manual pathology reporting costs, the confirmatory endoscopy, and the time of the nurse administering the test (see Appendix). Treatment costs include proton pump inhibitor and histamine 2 receptor antagonists drugs, endotherapy, esophagectomy, chemotherapy, and palliative care costs. Unit costs were taken from published sources [21,22]. Palliative care costs were applied to anyone who died of late (stage 4) EAC. We calculated mean and standard deviation costs for both arms for each cost component and all components combined (Table 3). Utilities and disutilities were derived from the literature (see Appendix) [20,[23], [24], [25]]. Disutilities were applied to stricture (2 weeks), perforation, EMR and RFA surgery (4 weeks), chemotherapy (4.5 months), and esophagectomy (3 months). The endoscopy costs were from UK tariffs which are likely to be an underestimate for private health care systems, and so higher endoscopy costs were explored in sensitivity analysis.

**Table 3:** Main results (per patient), showing the breakdown of costs and benefits for the intervention and usual care arms that make up the incremental cost-effectiveness ratio.

	Intervention arm		Usual care arm		
	Mean	SD	Mean	SD	Mean Difference
Screening cost	£77	£83	£1.14	£1.21	£76
Treatment cost	£489	£302	£482	£306	£7
Total cost	£565	£313	£48	£306	£82
QALYs gained	9.926	0.444	9.911	0.442	0.015
Life Years gained	13.027	0.545	13.016	0.545	0.011

ICER					£5500

Cost values given in GBP.

### 2.5 Transition probabilities, effectiveness and model structure

Natural history transition probabilities (se Appendix) were drawn from the literature [6,16,20]. The effectiveness of treatment for RFA and EMR was taken from published sources [7,26]. The effectiveness of esophagectomy was estimated by using 90-day mortality data taken from a National EAC audit [27]. We developed a new economic model, building on previous research into Cytosponge screening by Benaglia et al. [16] with adaptations to the model structure as well as parameters used for the natural history of BE and EAC treatment costs (Fig. 1,and Appendix).

### 2.6 Sensitivity analysis and budget impact

To explore the uncertainty around model parameters, we undertook probabilistic sensitivity analysis (PSA) and deterministic sensitivity analysis (DSA). PSA randomly and simultaneously varies all parameters within independent probability distributions, which we simulated 1000 times. The PSA results are illustrated using a cost-effectiveness plane, and a cost-effectiveness acceptability curve. The resulting ICER was presented with a 95% confidence interval.

DSA varies all parameters individually in order to determine the effect of each upon the ICER, identifying which parameters have the largest impact on cost-effectiveness. For the DSA, we varied the mean +/- 20% for all parameters, with the exception of: cytosponge uptake which was varied from 10% to 50%; the starting age of patients entering the model that was varied from 50 to 74 (ranging from the recommended starting age of screening to the upper quartile from the BEST3 trial data); prevalence of BE which was varied from 4% to 12%; Cytosponge cost which we varied from £144 to £344 per test; and Cytosponge sensitivity which we varied from 76.4% to 83.0% as per the BEST2 trial results [19].

Using the results of the analysis, we calculated the potential budget impact that adopting the Cytosponge-TFF3 would mean for large-scale role out within the NHS.

An alternative screening scenario, consistent with BEST3 was considered in which a second Cytosponge test was administered in a subset of patients following an inconclusive sample. 10% (202/1952) of Cytopsonge tests were re-administered in the BEST3 trial, and the impact of this on costs and QALYs was estimated in this scenario. This scenario also increases the sensitivity to >90% but we kept our conservative estimate of 79.9% [19].

### 2.7 Role of the funding source

The BEST3 trial was funded by Cancer Research UK, National Institute for Health Research, the UK National Health Service, Medtronic, and the Medical Research Council. Named authors had access to the data and decided to submit the manuscript for publication.

## 3 Results

### 3.1 Cost of screening

In the base-case analysis there were 1654 Cytosponge tests administered and 198 confirmatory endoscopies, giving a total cost of £524,716, or £77 per GERD patient. In the usual care arm, in which endoscopy was performed if deemed warranted by the family practitioner according to patient symptoms, there were 16 endoscopies performed (with biopsy) at a cost of £7808 or £1 per GERD patient.

### 3.2 Base-case analysis

The cost of one round of Cytosponge-TFF3 screening, including treatment for BE and EAC identified, and palliative care, was an incremental £82 per GERD patient compared with usual care. The Cytosponge arm generated an additional 0.015 QALYs per patient, and therefore the ICER was £5500 per QALY gained (Table 3). Patients gained on average 0.011 additional life years in the Cytosponge arm vs usual care.

### 3.3 Probabilistic sensitivity analysis

**Fig. 2:**
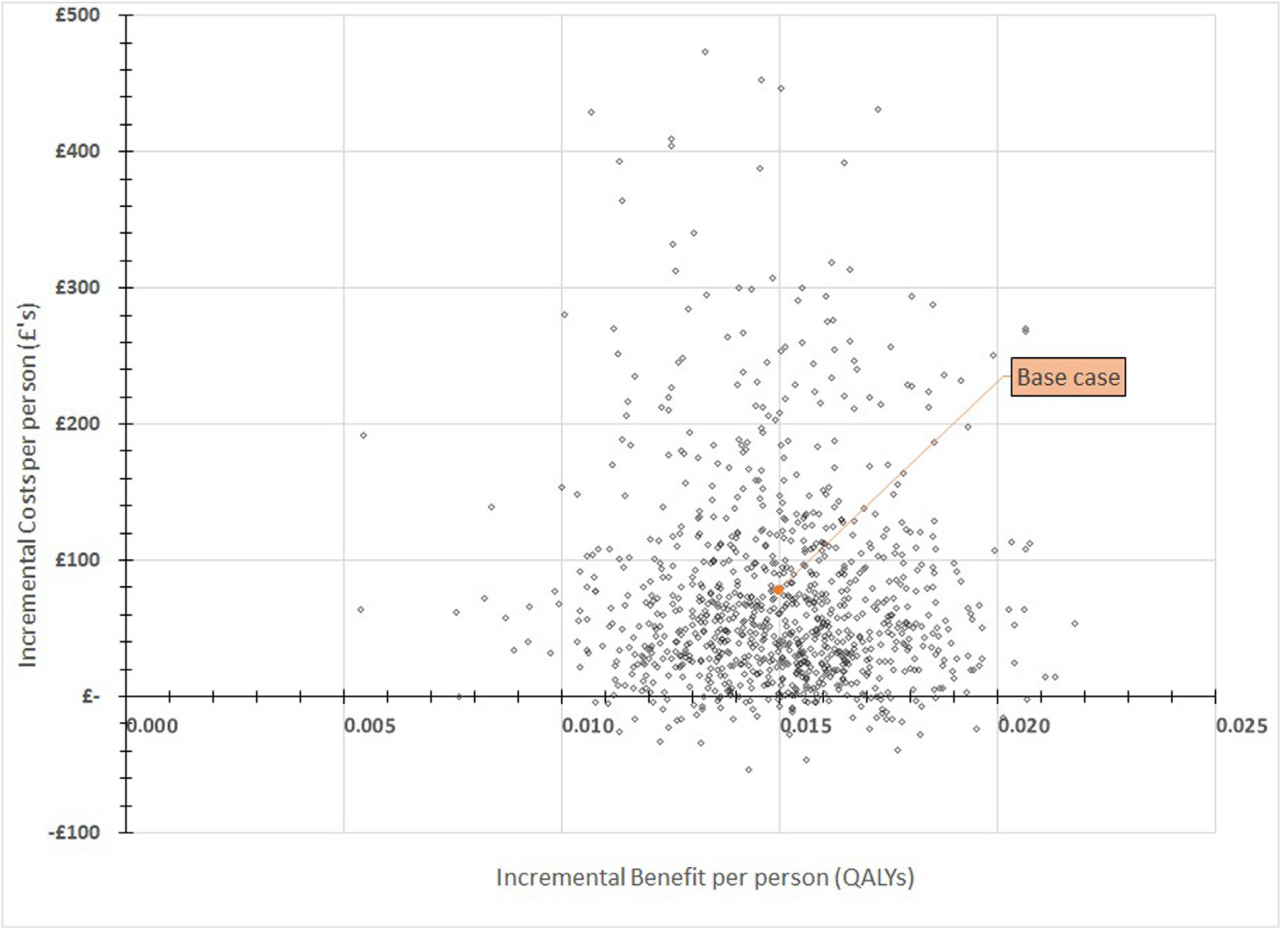
Cost-effectiveness plane. Each diamond represents the results of one simulation of the probabilistic sensitivity analysis in terms of per-person costs and quality-adjusted life-years (QALYs) gained. The base-case result of an incremental £81 and 0.015 QALYs is highlighted for reference. Graph shows incremental cost per person in GBP(£) on the y-axis and incremental QALYs on the *x*-axis.

**Fig. 3:**
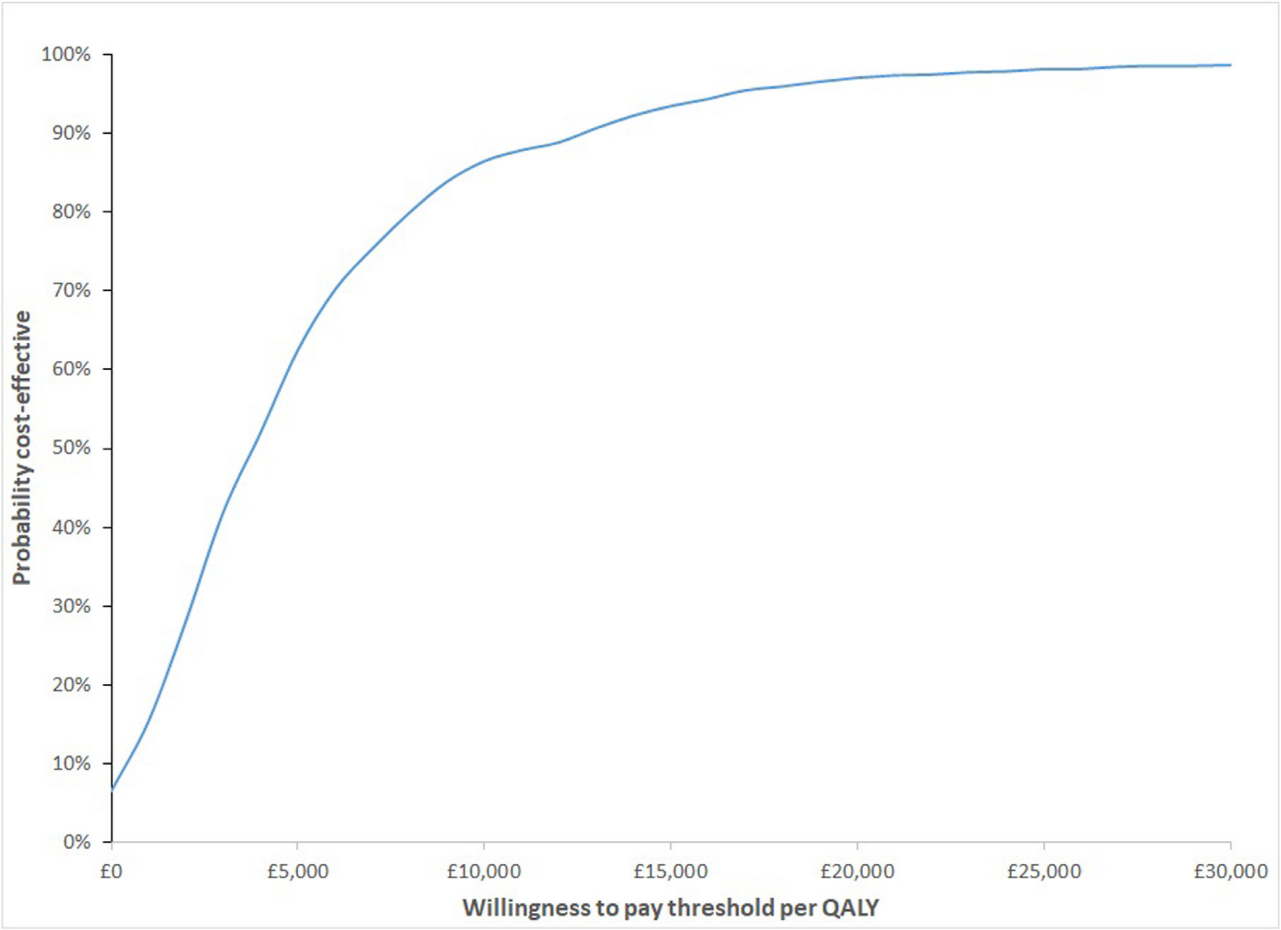
Cost-effectiveness acceptability curve. The curve illustrates the probability that the incremental cost-effectiveness ratios produced by the probabilistic sensitivity analysis are below the willingness-to-pay threshold of £20 000 per quality-adjusted life-year (QALY) gained recommended by the National Institute for Health and Care Excellence. Graph shows percentage change of being cost-effective on the y-axis and willingness-to-pay per QALY gained on the x-axis in GBP(£).

The PSA was used to estimate the uncertainty around the base-case estimates for the incremental mean cost per GERD patient and the incremental mean number of QALYs per GERD patient for both the Cytosponge and usual care arms. For the Cytosponge arm, these were an average of £582 (SD £313) and 9.92 QALYs (SD 0.44). For the usual care arm, these were an average of £504 (SD £306) and 9.91 QALYs (SD 0.44), which means an incremental cost of £78 (SD £86) and 0.015 QALYs (SD 0.002), giving an ICER of £5405 (95% CI −6791 to £17,600). At a willingness-to-pay threshold of £20,000 per QALY, the probability that the Cytosponge-TFF3 was cost-effective relative to usual care was 97% (Fig. 2, Fig. 3). The results of the 1000 PSA simulations are presented in Fig. 2, plotted on the cost-effectiveness plane. The likely cost-effectiveness of Cytosponge vs usual care at increasing thresholds of willingness-to-pay is plotted shown by the cost-effectiveness acceptability curve in Fig. 3. This suggests that Cytosponge screening is the more cost-effective option.

### 3.4 Deterministic sensitivity analysis

**Fig. 4:**
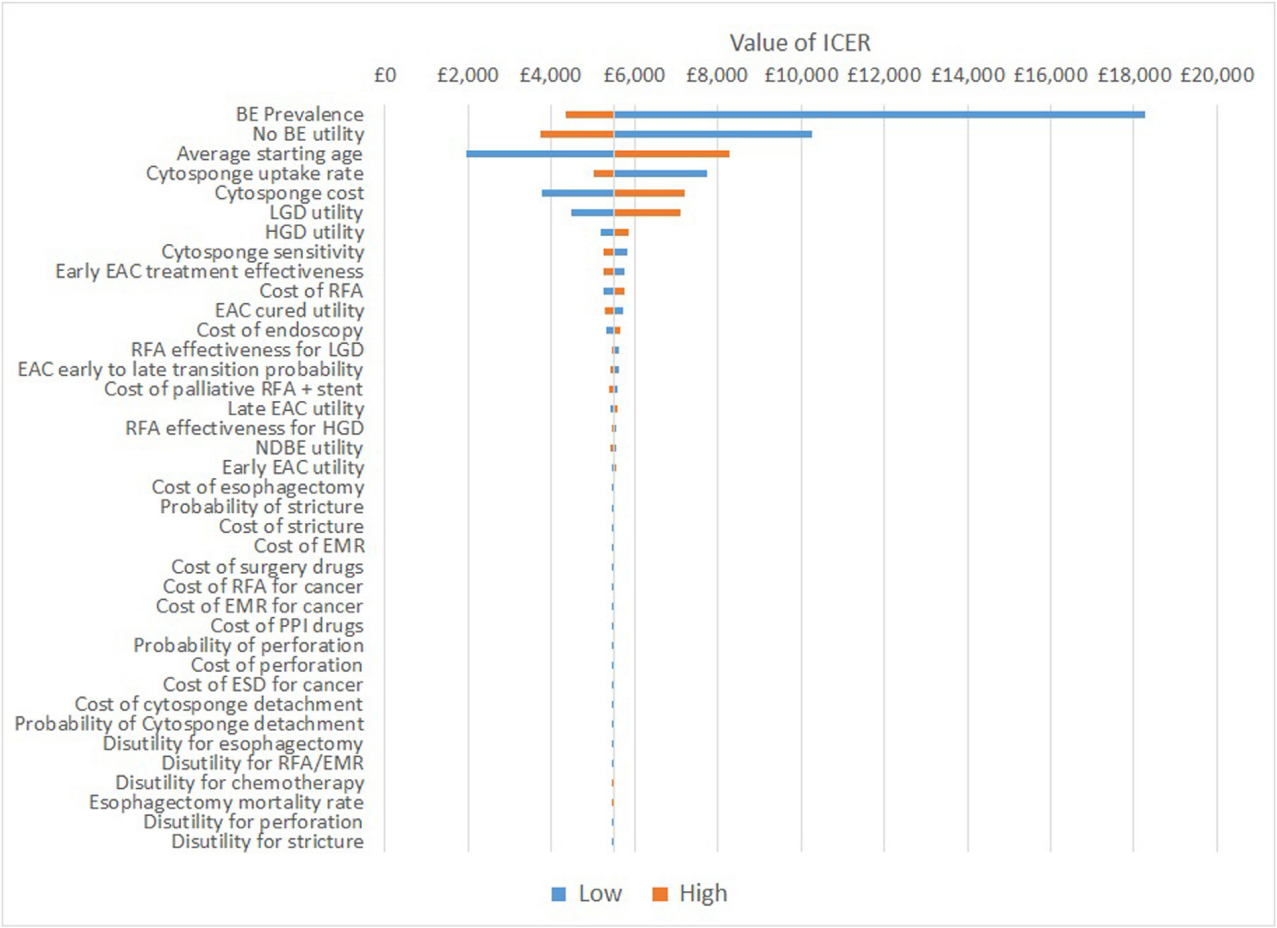
Tornado plot for the deterministic sensitivity analysis. Each parameter in the model is illustrated. The effect on the base case incremental cost-effectiveness ratio (ICER) of £5500 of reducing (low) or increasing (high) a given parameter can be observed in the ‘low’ and ‘high’ horizontal bars. BE = Barrett esophagus, LGD = low-grade dysplasia, EAC = esophageal. ICER values given in GBP(£).

The DSA shows that there were a number of parameters that had a large effect on the ICER, and these are presented and ranked on the tornado plot in Fig. 4. The parameters that had the largest impact were: the utility of the ‘No BE’ health state; the average starting age of the patients (at time *t* = 0); the prevalence of BE; the utility of the ‘LGD’ health state; the cost of Cytosponge and the uptake rate of Cytosponge.

Changing the utility of the health states ‘No BE’ and ‘LGD’ saw the ICER range from £3756 to £10,268 for ‘No BE’, and from £4488 to £7102 for ‘LGD’. That these parameters had a large impact is due to the fact that most patients in the model were in either of these health states at any given time (other than ‘dead’). Varying the average starting age saw the ICER range from £1952 to £8286. Varying the prevalence of BE saw the ICER range from £4352 to £18,256 (with a higher prevalence meaning Cytosponge is more cost-effective). Varying the cost of Cytosponge saw the ICER range from £3788 to £7212, with lower costs producing lower ICERs. Varying the uptake of Cytosponge saw the ICER range from £5008 to £7742, and at the upper value of 50% uptake the cost-per-patient was £173, along with fewer cases of HGD, EAC, and EAC mortality shown in Table 2.

### 3.5 Readministered cytosponge scenario

In the scenario where patients with inconclusive Cytosponge tests were invited for a repeat test, there were 202 additional Cytosponge tests administered and 23 additional confirmatory endoscopies. This resulted in identification of an additional 0.1 LGD, 1.0 HGD, 2.0 early EAC and 3.1 EAC deaths vs the base case analysis. The ICER was £5305, and the average cost of screening per person was £92.

### 3.6 Budget impact analysis

Using an estimated additional cost-per-patient of £82, we calculated that the total budget impact of conducting one round of Cytosponge-TFF3 screening in the UK would affect approximately 262,941 patients with GERD eligible for Cytosponge screening (assuming uptake of 24%) and would cost £21,636,235. This cost would be spread over roughly 29 years at an annual cost of £746,077.

## 4 Discussion

The introduction of Cytosponge-TFF3 screening is cost-effective relative to usual care, even at the relatively modest uptake of 24% and with a significantly older population than has been previously modelled. The base-case ICER of £5500 proved largely unresponsive to uncertainty around key parameters such as Cytosponge-TFF3 cost and endoscopy cost, increasing to a maximum of £18,256 per QALY gained in the deterministic sensitivity analysis (Fig. 4). This is a lower ICER than previously reported in Benaglia et al. [16] The favourable ICER is largely due to the fact that the usual care arm detected very few cases of BE relative to Cytosponge-TFF3, and since this model is underpinned with clinical trial data from a more real-world setting, we have much more confidence in these results.

As shown by the tornado plot, the cost-utility estimated by this model could be further improved by increasing the uptake of the Cytosponge-TFF3. The uptake is likely to be dependent on how the offer is made and inviting patients with symptoms at their general practitioner consultation or when ordering a repeat prescription is likely to make people more willing to take the test compared to an unexpected written invitation to take part in a trial. Simply because there are a comparatively large number of patients with NDBE, improving their health-related quality of life would also see a large improvement on the cost-effectiveness of Cytosponge-TFF3 screening. For example, patients with BE with the lowest risk of progression, i.e. with less than 2 cm of segment length, younger age, female sex [28], do arguably not require regular surveillance and clinical guidelines are likely to reflect this in coming years. Work is ongoing so that additional risk stratification biomarkers applied to the Cytosponge-TFF3 test will enable monitoring to be performed non-endoscopically [19].

It ought to be noted that the absolute gain in QALYs is small because the majority of patients in the model will not develop BE and therefore will have no resulting change in their utility (which is based on each disease state). However, for the small number of patients who are detected early and treated the QALY gain is substantial, and indeed the utility gain is greater than the length of life gained through reduced mortality (demonstrated by the fact that the QALY gain is larger than the life years gain between the arms).

Strengths of this analysis include the use of data from a real-world clinical trial, such that it was not reliant upon estimates of prevalence and incidence which often have a large degree of uncertainty around them. We used conservative estimates for the Cytosponge-TFF3 device and laboratory costs, which are likely to come down as manufacturing and processing throughput increases. Using the sensitivity and specificity of a BE screening tool to estimate the false negatives, coupled with real-world data on all true positives, should give a robust estimate of the true prevalence of BE in GERD patients. Additionally, our method of estimating prevalence yielded a similar proportion of cases of BE to that of other published work [16]. We also point out that we took endoscopy, the gold standard, to be 100% accurate which we know is not the case with increasing data on post-endoscopy esophageal adenocarcinoma suggesting a 3–13% miss rate [29].

Limitations include the lack of available data on transition probabilities between stages of EAC, and lack of available data needed to estimate the standard errors of our PSA parameters. We have tried to overcome this by using values that will bias against the intervention. Updating this analysis with treatment transition probabilities for NDBE would be a recommendation for further study. Additionally, the model predicts 20 fewer EAC deaths in the Cytosponge arm (although this was not the model's primary function and we lacked robust data on EAC progression risk) and again this would be affected by the uptake of the test (Table 2). Microsimulation models are required to evaluate this further. Lastly, the median age from BEST3 used here is for those who took the Cytosponge test and is skewed by more elderly persons accepting a postal invitation offer for research and having time to attend a trial test. A screening program targeting slightly younger participants will likely see additional benefit as younger participants will accrue more QALYs as they stay in better health states for longer. Similarly, a screening intervention focusing on male patients is likely to have a positive effect on the ICER, considering the comparatively higher risk of BE progression in males [28] and therefore the potential gain in quality and length of life from early diagnosis and treatment.

This analysis considered only one round of screening for one cohort. Continued monitoring of the BEST3 cohort will allow this analysis to be updated when the post-screening incidence of BE can be identified in the BEST3 cohort, and future economic modeling will help to inform whether a program of screening every 3, 5 or 10 years, for example, would be more cost-effective. In addition, as evidence accrues on predictive risk scores for BE and EAC, this may help to identify the optimum group for targeted screening strategies to be cost-effective [30], including considering enriching the population at risk without reliance on a history of reflux [31].

In conclusion, the BEST3 trial showed that an offer of Cytosponge-TFF3 screening was very effective at identifying cases of BE and EAC relative to identification of BE by endoscopy based on referrals. This economic evaluation has shown that the Cytosponge-TFF3 yielded modest benefits at a low cost compared to usual care. This is largely because the majority of patients screened did not have BE, and most of the cases detected tended to be milder cases (NDBE), which incur relatively little cost. Except for the upfront screening cost, the total cost of one round of Cytosponge-TFF3 screening would likely be spread over many years as most cases of BE found were of low severity and relatively stable. These data are encouraging for the rapidly expanding research efforts to develop non-endoscopic screening strategies for Barrett esophagus [32], [33], [34] and paves the way for further modeling studies to evaluate cost-effectiveness and health benefits in a range of health care systems.

## Data sharing

The trial is a cluster randomised trial with aggregated data for the usual care arm and therefore individual level data will only be available for the Cytosponge intervention patients. GP-level data and individual data will be available via the University of Cambridge's data repository (https://www.data.cam.ac.uk/repository). The BEST3 website (https://www.best3trial.org/) is available for more information and contact details.

## Declaration of Competing Interest

BM reports employment contract by Cyted, outside the submitted work. PS reports grants from Cancer Research UK and Innovate UK, during the conduct of the study; consulting fees from GRAIL Inc, grants from NIHR, Yorkshire Cancer Research and Public Health England, and being in an advisory committee for NHS England on use of Cytosponge (unpaid) and in one on NCRI screening, prevention and Early Detection (unpaid) outside the submitted work. RM reports payment made to Cancer Research UK. RCF reports grants from Cancer Research UK, during the conduction of the study, and has a patent Cytosponge TM co-inventor licensed to Medtronic by the Medical Research Council, reports fees from presentation by Medtronic, is an Editor at the American Association Journal of Gastroeneteroly and co-founder and share-holder of Cyted Ltd an early detection company, outside the submitted work. SM & NS reports grants from Cancer Research UK and from Innovate UK, during the conduct of the study.

## Appendix Supplementary materials

Image, application 1
