# Supplementary material for: Economic evaluation of Cytosponge®-trefoil factor 3 for Barrett esophagus: A cost-utility analysis of randomised controlled trial data
Source: eClinicalMedicine. 2021 Jun 18;37:100969. doi: 10.1016/j.eclinm.2021.100969 (PMC8225801; doi:10.1016/j.eclinm.2021.100969)
Supplement: Supplementary file 1 [file mmc1.docx]

Appendix

# Methods (Extended)

## Estimating disease prevalence

To estimate the prevalence of BE in this cohort, we used the sensitivity of the Cytosponge-TFF3 test to BE when incorporating repeat testing for those with an inadequate test. This was 89.7% (95% CI 82.3%-94.8%) in the BEST2 case-control study, designed for accuracy as a primary endpoint^1^, along with the number of cases of BE identified by successful Cytosponge-TFF3 tests and confirmed by endoscopy with biopsy (i.e. the true positive cases) from BEST3^2^ (n = 127). We assumed that prevalence is the same in those who did and did not have a Cytosponge-TFF3 test. Using these values in combination with the number of true positive cases and the uptake rate of Cytosponge-TFF3 (24%) allowed us to estimate the number of false negatives, and the prevalence of all BE of 8.6% (142/1654), i.e. Cytosponge-TFF3 ‘missed’ an estimated 15 cases of BE. This prevalence figure is similar to estimates from Rubenstein et al.^3^ We put the false negative cases in the ‘Natural history’ model starting at the appropriate stage of disease (i.e. NDBE, Low-Grade Dysplasia (LGD), High-Grade Dysplasia (HGD), early or late EAC). The true negatives and false positives went into the Treatment model, starting in the ‘No BE’ state (false positives having incurred the screening cost of Cytosponge-TFF3 plus endoscopy). Therefore, the majority of patients were in the Natural history model of both study arms, either because they were in the usual care arm and were not referred for an endoscopy by their general practitioner, or because they did not take up the offer of Cytosponge-TFF3 screening. The latter was under the assumption that such participants were not referred for an endoscopy by their general practitioner. We assumed that the confirmatory endoscopy with biopsy that follows a positive TFF3 test result had an effective sensitivity and specificity of 1 (gold-standard) for the purposes of the model. This consideration takes into account the face validity of a negative endoscopy test in that clinicians typically do not re-order endoscopy following negative findings, even though the sensitivity of endoscopy is less than 100%.

## EAC treatment

Treatments given for early and late-stage EAC were taken from trial data. Treatments recorded for early-stage EAC were a combination of radiofrequency ablation (RFA), endoscopic mucosal resection (EMR), endoscopically submucosal dissection, argon plasma coagulation, chemotherapy and oesophagectomy. Treatments recorded for late-stage EAC were a combination of palliative radiotherapy, stent and chemotherapy. There were nine individuals with dysplastic BE or stage-I oesophagogastric cancer in the intervention arm and none in the usual care arm. Of these, eight were detected as a result of a positive Cytosponge-TFF3 which triggered an endoscopy and they all underwent a curative intervention (seven endoscopic therapies, one oesophagectomy for a stage-IB (sm1) cancer), and the ninth case was a patient who initially expressed interest but was referred for an endoscopy by their general practitioner before having a Cytosponge-TFF3 examination. In addition, there were five stage II or worse cancers diagnosed in the trial: three in the usual care arm and two in the intervention arm amongst patients who did not undergo the Cytosponge-TFF3 test.

No intervention was given to anyone diagnosed with NDBE since, according to National society guidelines, treatment is only given if dysplasia or early EAC is found. Late stage cancer is treated with palliative care. Utilities were assigned regardless of whether any disease has been identified. Key model outputs are given in Table 2.

## Utility

Utilities and disutilities were derived from the literature (Table 3)^4–7^. Whereas NICE CG106^4^ used a value of 1 for the utility of “No BE”, we used the EuroQoL UK population norms^8^ for age group 65-74 of 0.779 for the “No BE” state. For each other health state, we took the difference in utility reported by NICE and subtracted that from our “No BE” utility. However, we felt it was unrealistic for early EAC to have the same utility as HGD, and therefore used the midpoint between HGD and late EAC as the utility for early EAC. Disutilities were applied to stricture (2 weeks), perforation, EMR and RFA surgery (4 weeks), chemotherapy (4.5 months), and oesophagectomy (3 months).

## Transition probabilities, effectiveness and safety parameters

Natural history transition probabilities were drawn from the literature^5,9,10^ and adapted for this analysis. Where possible, we used the values from Benaglia et al^9^ (the previous economic evaluation of Cytosponge-TFF3) based on Garside et al^11^. Further transition data was drawn from Pollit et al^5^ adapting values from Inadomi et al^10^. Inadomi used adjusted rates to fit overall cancer incidence statistics, and Pollit’s adjustment of those values are more recent and designed for a model that is more similar to the one we developed here. We assumed that late (stage 4) EAC always transitioned to “Dead (EAC)” within 1 cycle (year).

## Cost-utility analysis

Cost-effectiveness was measured in terms of an incremental cost-effectiveness ratio (ICER) – the incremental cost per quality-adjusted life-year (QALY) gained. The costs for the Intervention arm were calculated as follows:

$$Cost of \mathrm{Intervention}arm={Cost}_{(Treatment model)}+{Cost}_{\left( Natural history model \right)}+ {Cost}_{(Screening)}$$

Where:

${Cost}_{(Treatment model)}$ is the cost of treatment, complications and palliative care for all patients in the Treatment model

${Cost}_{\left( Natural history model \right)}$ is the cost of treatment, complications and palliative care for all patients in the Natural history model

${Cost}_{(Screening)}$ is the cost of Cytosponge-TFF3 screening for the cohort, including the Cytosponge-TFF3 device, laboratory costs (including: sample processing, antibody staining, pathology reporting time), nurse time to administer Cytosponge-TFF3, confirmatory endoscopies with biopsy, and potential Cytosponge-TFF3 detachments.

A similar calculation was used for the usual care arm, except that screening cost did not include Cytosponge-related costs but only the costs arising from any endoscopy referrals by a general practitioner. QALYs were calculated based on the utilities of each health state multiplied by the number of people in each health state per cycle (year), minus the utility decrements of complications of treatment.

## BEST3 Consortium

The Trial was managed by the Cancer Research UK & King’s College London Cancer Prevention Trials Unit. The BEST3 consortium consists of the following:

Independent Data monitoring committee: Stephen Attwood (Chair), Max Parmar, Brendan Delaney. Independent Trial steering committee: John de Caestecker (Chair), Wendy Atkin (in memoriam), Allan Hackshaw, Charles van Heyningen (patient representative), Tim Underwood

Cancer Prevention Trials Unit: Alberto Stella, Charlotte Saxby, Attila Lorincz, Naomi Turnbull, Jamie Doorbar, Georgia Mannion-Krase, Irene Kaimi (in memoriam)

Cambridge University Hospitals/Cambridge University: Staff at the Cambridge University Hospitals Human Research Tissue Bank, Mary Kasanicki, Stephen Kelleher, Louise Stockley, Tracy Assari, Sonakshi Kadyan, Victoria Hollamby, Katie Edwards CRN Eastern: Helen MacDonald, Viv Shaw, Heather Leishman, Holly Roper, Kate McCloskey, Helen Jung, Alex Phillips, Gosia Masjak-Newman, Kim Fell and the delivery team CRN Thames Valley and South Midlands: Helen Collins, Olga Zolle, and study delivery team CRN South West Peninsula: Pauline McGlone, Tania Crabb, Lauren Merrin and study delivery teams. CRN Wessex: Martine Cross, Alex Jones, Tom Simpson and study delivery team CRN North East and North Cumbria: Emma Murray and study delivery teams. CRN Yorkshire and Humber/Yorkshire practices: Study delivery teams. CRN North Thames: Andrew Perugia and study delivery team CRN East Midlands and University of Nottingham: Marie Thompson, Jen Dumbleton, Monique Morar and Nadia Frowd. Participating NHS trusts: Antonia Hardcastle, Debbie Carmichael, Fiona Maxton, Frances Farnworth, Elaine Baddeley

# All Model Parameters

| **Model Parameter** | **Mean** | **SE** | **Distribution** | **alpha** | **beta** | **Source** |
| --- | --- | --- | --- | --- | --- | --- |
| **Efficiency/Safety** |  |  |  |  |  |  |
| Cytosponge-TFF3 Sensitivity with repeat test | 0.799 | 0.017 | Beta | 451.9 | 113.7 | Ross-Innes 2015^1^ |
| Cytosponge-TFF3 Specificity | 0.93 | 0.013 | Beta | 341.3 | 25.5 | Ross-Innes 2015^1^ |
| RFA for LGD | 0.93 | - | Beta | 63 | 5 | Phoa 2014^12^ |
| RFA for HGD | 0.93 | - | Beta | 50 | 4 | Shaheen 2011^13^ |
| Treatment for early EAC | 0.85 | 0.085 | Beta | 14.2 | 2.5 | Assumption based on expert opinion |
| Cytosponge-TFF3 Detachments | 0.0005 | - | Beta | 1.0 | 1843 | BEST3 Trial Data |
| RFA Stricture | 0.056 | 0.008 | Beta | 44.4 | 748 | Qumseya 2016^14^ |
| RFA Perforation | 0.006 | 0.001 | Beta | 22.0 | 3643 | Qumseya 2016^14^ |
| Esophagectomy 90-day mortality | 0.033 | 0.003 | Beta | 96.7 | 2833 | NOGCA annual report 2017^15^ |
| EAC T1/2 patients receiving treatment |  |  |  |  |  |  |
| Proportion receiving RFA | 0.25 | 0.16 | Beta | 4 | 1 | BEST3 Trial Data |
| Proportion receiving EMR | 0.75 | 0.17 | Beta | 4 | 3 | BEST3 Trial Data |
| Proportion receiving APC | 0.25 | 0.16 | Beta | 4 | 1 | BEST3 Trial Data |
| Proportion receiving ESD | 0.25 | 0.16 | Beta | 4 | 1 | BEST3 Trial Data |
| Proportion receiving Esophagectomy | 0.50 | 0.18 | Beta | 4 | 2 | BEST3 Trial Data |
| EAC T3/4 patients receiving treatment |  |  |  |  |  |  |
| Proportion receiving chemo | 0.25 | 0.16 | Beta | 4 | 1 | BEST3 Trial Data |
| Proportion receiving palliative RFA and stent | 0.75 | 0.17 | Beta | 4 | 3 | BEST3 Trial Data |
| Prevalence of Barrett esophagus | 0.09 | 0.02 | Beta | 20 | 187 | Assumption based on BEST3 Trial Data |
| **Unit Costs** |  |  |  |  |  |  |
| *Surveillance* |  |  |  |  |  |  |
| Cytosponge device, processing, TFF3 immunostaining and reporting | £230 | £230 | Gamma | 1 | 230 | NHS acquisition price. |
| Nurse time administering Cytosponge | £14 | £14 | Gamma | 1 | 14 | PSSRU 2019 - Nurse (GP Practice)^16^. Trial data indicates average Cytosponge-TFF3 test time is 20 mins. |
| Confirmatory endoscopy without biopsy | £430 | £430 | Gamma | 1 | 430 | National Schedule of NHS Costs 2018/19^17^. Diagnostic Endoscopic Upper Gastrointestinal Tract Procedures, 19 years and over. FE21Z. Day Case. |
| Confirmatory endoscopy with biopsy | £488 | £488 | Gamma | 1 | 488 | National Schedule of NHS Costs 2018/19^17^. Diagnostic Endoscopic Upper Gastrointestinal Tract Procedures with biopsy, 19 years and over. FE21Z. Day Case. |
| Cytosponge detachment | £1,084 | £1,084 | Gamma | 1 | 1084 | National Schedule of NHS Costs 2018/19^17^. Therapeutic Endoscopic Upper Gastrointestinal Tract Procedures, 19 years and over. FE21Z. Non-elective. |
| *Treatment* |  |  |  |  |  |  |
| PPI drugs (annual) | £22 | £22 | Gamma | 1 | 22 | Weighted average of drugs and dosages taken from BEST3 trial data. |
| PPI drugs following surgery | £45 | £45 | Gamma | 1 | 45 | Assumption based on expert opinion. |
| H2 antagonist drugs | £426 | £426 | Gamma | 1 | 426 | BNF - assumed one year of effervescent ranitidine. |
| RFA | £3,414 | £3,414 | Gamma | 1 | 3414 | National Schedule of NHS Costs 2018/19^17^. Upper GI surgery. FE20Z. Therapeutic Endoscopic Upper Gastrointestinal Tract Procedures, 19 years and over. Elective. Assumed average of 3 treatments (£379 per treatment). |
| EMR | £1,138 | £1,138 | Gamma | 1 | 1138 | National Schedule of NHS Costs 2018/19^17^. Upper GI surgery. FE02Z. Therapeutic Endoscopic Upper Gastrointestinal Tract Procedures, 19 years and over. Elective. |
| RFA cancer | £3,748 | £3,748 | Gamma | 1 | 3748 | NICE guidelines for OAC management 2018^18^ |
| ESD | £1,423 | £1,423 | Gamma | 1 | 1423 | Assumption based on expert opinion. (ESD is a longer more complicated EMR. Therefore cost = 25% more than EMR.) |
| Chemotherapy | £3,299 | £3,299 | Gamma | 1 | 3299 | NICE guidelines for OAC management 2018^18^ |
| Esophagectomy | £9,433 | £9,433 | Gamma | 1 | 9433 | National Schedule of NHS Costs 2018/19^17^. Weighted average: Complex, Oesophageal, Stomach or Duodenum Procedures, 19 years and over. Elective inpatient (6 days stay). |
| Stricture | £4,905 | £4,905 | Gamma | 1 | 4905 | National Schedule of NHS Costs 2018/19^17^. Weighted Average: Major Therapeutic Endoscopic, Upper or Lower Gastrointestinal Tract Procedures, 19 years and over. Non-elective inpatient (non-elective because adverse event of treatment) and Day case. |
| Perforation | £7,554 | £7,554 | Gamma | 1 | 7554 | National Schedule of NHS Costs 2018/19^17^. Weighted average: Complex, Oesophageal, Stomach or Duodenum Procedures, 19 years and over. Elective inpatient. |
| Palliative Stent | £5,320 | £5,320 | Gamma | 1 | 5320 | National Schedule of NHS Costs 2018/19^17^. FEC10. Endoscopic Insertion of Luminal Stent into Gastrointestinal Tract with CC Score 7+. Elective Inpatient. |
| Palliative care | £7,287 | £7,287 | Gamma | 1 | 7287 | Georghiou 2014, cited in NICE guidelines for OAC management 2018^18^. |
| **Utility (annual)** |  |  |  |  |  |  |
| *Health states* |  |  |  |  |  |  |
| ‘No BE’ | 0.779 | 0.04 | Beta | 87.6 | 24.9 | NICE CG106, Appendix 6^4^. Adjusted. SE is the average of other SE's used. |
| NDBE | 0.689 | 0.03 | Beta | 123.7 | 55.8 | NICE CG106, Appendix 6^4^. Adjusted. |
| LGD | 0.629 | 0.03 | Beta | 147.8 | 87.2 | NICE CG106, Appendix 6^4^. Adjusted. |
| HGD | 0.549 | 0.03 | Beta | 179.9 | 147.7 | NICE CG106, Appendix 6^4^. Adjusted. |
| EAC T1/2 | 0.502 | 0.03 | Beta | 198.9 | 197.7 | Assumption. Mean is midpoint between HGD and OAC/T3. SE assumed the same as HGD. |
| EAC T3/4 | 0.454 | 0.02 | Beta | 217.9 | 262.1 | NICE CG106, Appendix 6^4^. Adjusted. |
| EAC - cured | 0.642 | 0.03 | Beta | 142.6 | 79.5 | Assumption. |
| *Treatment*/*complication* |  |  |  |  |  |  |
| Stricture | - 0.03 | - 0.002 | Gamma | 400.00 | - 0.000 | NICE CG106, Appendix 6^4^. Adjusted. |
| Surgery for perforation | - 0.28 | - 0.014 | Gamma | 400 | - 0.001 | NICE CG106, Appendix 6^4^. Adjusted. |
| EMR and RFA surgery | - 0.06 | - 0.003 | Gamma | 400 | - 0.000 | Pollit et al^5^ |
| Chemotherapy | - 0.09 | - 0.005 | Gamma | 400 | - 0.000 | Doherty et al^7^ |
| Esophagectomy surgery | - 0.26 | - 0.013 | Gamma | 400 | - 0.001 | NICE CG106, Appendix 6^4^. Adjusted. |
| **Transition probabilities** |  |  |  |  |  |  |
| ‘No BE’ to NDBE | 0.005 | 0.002 | Beta | 6.0 | 1188 | Inadomi et al (2009)^10^. Adjusted by Benaglia et al. (2013)^9^ |
| NDBE to LGD | 0.029 | 0.003 | Beta | 68.4 | 2297 | Inadomi et al (2009)^10^. Adjusted by Benaglia et al. (2013)^9^ |
| NDBE to HGD | 0.005 | 0.001 | Beta | 99.4 | 17 981 | Inadomi et al (2009)^10^. Adjusted by Pollit et al. (2018) ^5^ |
| NDBE to EAC | 0.003 | 0.0003 | Beta | 99.7 | 35 514 | Inadomi et al (2009)^10^. Adjusted by Pollit et al. (2018) ^5^ |
| LGD to HGD | 0.028 | 0.003 | Beta | 97.2 | 3438 | Inadomi et al (2009)^10^. Adjusted by Pollit et al. (2018) ^5^ |
| LGD to EAC (early) | 0.014 | 0.001 | Beta | 98.6 | 7047 | Inadomi et al (2009)^10^. Adjusted by Pollit et al. (2018) ^5^ |
| HGD to EAC (early) | 0.119 | 0.012 | Beta | 88.0 | 653 | Inadomi et al (2009)^10^. Adjusted by Benaglia et al. (2013) ^9^ |
| EAC (early) to EAC (late) | 0.800 | 0.080 | Beta | 19.2 | 5 | Assumption based on expert opinion from the BEST3 team |
| *Disutilities were applied for a duration based on expert opinion: stricture – two weeks; surgery for perforation, EMR and RFA – four weeks; chemotherapy – eighteen weeks; esophagectomy – three months.* | | | | | | |

## References

1. Ross-Innes CS, Debiram-Beecham I, O’Donovan M, Walker E, Varghese S, Lao-Sirieix P, et al. Evaluation of a Minimally Invasive Cell Sampling Device Coupled with Assessment of Trefoil Factor 3 Expression for Diagnosing Barrett’s Esophagus: A Multi-Center Case–Control Study. Franco EL, editor. PLOS Med. 2015 Jan 29;12(1):e1001780.

2. Fitzgerald RC, Pietro M di, O’Donovan M, Maroni R, Muldrew B, Debiram-Beecham I, et al. Cytosponge-trefoil factor 3 versus usual care to identify Barrett’s oesophagus in a primary care setting: a multicentre, pragmatic, randomised controlled trial. The Lancet. 2020 Aug 1;396(10247):333–44.

3. Rubenstein JH, Inadomi JM, Brill JV, Eisen GM. Cost utility of screening for Barrett’s esophagus with esophageal capsule endoscopy versus conventional upper endoscopy. Clin Gastroenterol Hepatol Off Clin Pract J Am Gastroenterol Assoc. 2007 Mar;5(3):312–8.

4. NICE. Barrett’s oesophagus - ablative therapy (CG 106) Appendix 6: cost effectiveness analysis for Barrett’s Oesophagus. 2010.

5. Pollit V, Graham D, Leonard C, Filby A, McMaster J, Mealing SJ, et al. A cost-effectiveness analysis of endoscopic eradication therapy for management of dysplasia arising in patients with Barrett’s oesophagus in the United Kingdom. Curr Med Res Opin. 2019 May 4;35(5):805–15.

6. Gerson LB, Ullah N, Hastie T, Goldstein MK. Does cancer risk affect health-related quality of life in patients with Barrett’s esophagus? Gastrointest Endosc. 2007 Jan;65(1):16–25.

7. Doherty MK, Leung Y, Su J, Naik H, Patel D, Eng L, et al. Health utility scores from EQ-5D and health-related quality of life in patients with esophageal cancer: a real-world cross-sectional study. Dis Esophagus [Internet]. 2018 Dec 1 [cited 2020 Mar 30];31(12). Available from: https://academic.oup.com/dote/article/doi/10.1093/dote/doy058/5037798

8. Szende A, Janssen B, Cabases J, editors. Self-Reported Population Health: An International Perspective based on EQ-5D [Internet]. Dordrecht: Springer Netherlands; 2014 [cited 2020 Aug 6]. Available from: http://link.springer.com/10.1007/978-94-007-7596-1

9. Benaglia T, Sharples LD, Fitzgerald RC, Lyratzopoulos G. Health Benefits and Cost Effectiveness of Endoscopic and Nonendoscopic Cytosponge Screening for Barrett’s Esophagus. Gastroenterology. 2013 Jan;144(1):62-73.e6.

10. Inadomi JM, Somsouk M, Madanick RD, Thomas JP, Shaheen NJ. A Cost-Utility Analysis of Ablative Therapy for Barrett’s Esophagus. Gastroenterology. 2009 Jun;136(7):2101-2114.e6.

11. Garside R, Pitt M, Somerville M, Stein K, Price A, Gilbert N. Surveillance of Barrett’s oesophagus: exploring the uncertainty through systematic review, expert workshop and economic modelling. Health Technol Assess. 2006;10(8).

12. Phoa KN, van Vilsteren FGI, Weusten BLAM, Bisschops R, Schoon EJ, Ragunath K, et al. Radiofrequency Ablation vs Endoscopic Surveillance for Patients With Barrett Esophagus and Low-Grade Dysplasia: A Randomized Clinical Trial. JAMA. 2014 Mar 26;311(12):1209.

13. Shaheen NJ, Overholt BF, Sampliner RE, Wolfsen HC, Wang KK, Fleischer DE, et al. Durability of Radiofrequency Ablation in Barrett’s Esophagus With Dysplasia. Gastroenterology. 2011 Aug;141(2):460–8.

14. Qumseya BJ, Wani S, Desai M, Qumseya A, Bain P, Sharma P, et al. Adverse Events After Radiofrequency Ablation in Patients With Barrett’s Esophagus: A Systematic Review and Meta-analysis. Clin Gastroenterol Hepatol Off Clin Pract J Am Gastroenterol Assoc. 2016;14(8):1086-1095.e6.

15. Varagunam M, Brand C, Cromwell D, Maynard N, Crosby T, Michalowski J, et al. National Oesophago-Gastric Cancer Audit Annual Report 2017. NOGCA. 2017;103.

16. CURTIS L. UNIT COSTS OF HEALTH AND SOCIAL CARE 2019. [S.l.]: UNIVERSITY OF KENT AT CAN; 2019.

17. NHS Improvement. National Schedule of NHS costs. NHS; 2019.

18. National Guideline Alliance (Great Britain), National Institute for Health and Care Excellence (Great Britain), Royal College of Obstetricians and Gynaecologists (Great Britain). Oesophago-gastric cancer: assessment and management in adults [Internet]. NICE; 2018 [cited 2020 Aug 6]. Available from: https://www.ncbi.nlm.nih.gov/books/NBK481429/
